# Severe Rhabdomyolysis as Complication of Interaction between Atorvastatin and Fusidic Acid in a Patient in Lifelong Antibiotic Prophylaxis: A Dangerous Combination

**DOI:** 10.1155/2016/4705492

**Published:** 2016-12-27

**Authors:** Anirban Nandy, Shahin Gaïni

**Affiliations:** ^1^Medical Department, Infectious Diseases Division, National Hospital Faroe Islands, Tórshavn, Faroe Islands; ^2^Infectious Diseases Research Unit, Odense University Hospital and University of Southern Denmark, Odense, Denmark; ^3^Faculty of Science and Technology, University of the Faroe Islands, Tórshavn, Faroe Islands

## Abstract

Atorvastatin and HMG-CoA reductase inhibitors are the most frequently used medication in the world due to very few adverse toxic side effects. One potentially life threatening adverse effect is caused by clinically significant statin induced rhabdomyolysis, either independently or in combination with fusidic acid. The patient in our case who previously had cardiac insufficiency, atrial fibrillation, and thoracic aorta aneurysm and was treated with insertion of an endovascular metallic stent in the aorta is presented in the report. He had an inoperable aortitis with an infected stent and para-aortic abscesses with no identified microorganism. The patient responded well to empirical antibiotic treatment with combination therapy of fusidic acid and moxifloxacin. This treatment was planned as a lifelong prophylactic treatment. The patient had been treated with atorvastatin for several years. He developed severe rhabdomyolysis when he was started on fusidic acid and moxifloxacin. The patient made a fast recovery after termination of treatment with atorvastatin and fusidic acid. We here report a life threatening complication of rhabdomyolysis that physicians must be aware of. This can happen either in atorvastatin monotherapy or as a complication of pharmacokinetic interaction between atorvastatin and fusidic acid.

## 1. Introduction

Rhabdomyolysis is most commonly described by “lysis” or disintegration and breakdown of skeletal muscles and subsequent release of toxic intracellular components into the systemic circulation. The major causes of rhabdomyolysis include trauma, infections, hyperthermia, myopathies, and adverse drug-drug interactions of certain medications [1]. Statin myotoxicity is a well-known side effect and is related to serum levels of the drugs and can also be influenced by coprescription with other drugs, thus increasing the risk of side effects including rhabdomyolysis [2–4]. Although it has been reported previously that there is an increased risk of myopathy with coprescription of atorvastatin and fusidic acid, it was not before 2011 that the avoidance of coprescription of this drug combination was recommended [5–7]. We present a patient already on statin treatment who developed rhabdomyolysis apparently precipitated by fusidic acid. The potential life threatening interaction between atorvastatin and fusidic acid is highlighted in this case report.

## 2. Case Presentation

A Caucasian man aged 75 years presented to the Emergency Department at the National Hospital, Faroe Islands, with complaints of a two-week history of progressively severe diffuse myalgia, confined to the lower back and proximal lower muscles, reduced power in all four extremities, and generalized weakness, and he was immobile and bedridden. He was neurologically sound and there was no complaint regarding headache, numbness, tingling, or paraesthesia. He denied indulgence in any kind of vigorous physical exercise and over consumption of alcohol. He was already under medication for hypertension and in treatment with rivaroxaban due to atrial fibrillation and atorvastatin 80 mg daily for hyperlipidaemia and on lifelong prophylactic empirical antibiotic treatment with oral fusidic acid 500 mg twice a day combined with oral moxifloxacin 400 mg daily and for an infected aortic aneurysm, with an infected endovascular aortic stent and para-aortic abscesses. He had no familial or prior personal history of muscle disorder and no past history of muscular toxicity with statin or fibrate use. He had been in treatment with atorvastatin for several years. He had been treated with fusidic acid and moxifloxacin for 6 days before the day of admission.

He was afebrile with heart rate 75 beats/minute but hypotensive (103/42 mmHg). The cardiovascular, respiratory, and gastrointestinal tract examination was normal. He complained of moderate discomfort due to myalgia. The quadriceps muscles were tender to palpation and he had reduced power bilaterally. The cranial nerves 2–12 examination was normal. The power 5/5 strength in all the muscle groups was observed, except for the hip flexors and quadriceps which rated 3/5 bilaterally. He was immobile due to weakness. He had normal sensory examinations to light touch, pin prick, and proprioception.

On admission the laboratory measurements revealed the following: hemoglobin 6.4 mmol/L, total leukocyte count 7.8 × 10^9^/L, potassium 4.4 mmol/L, sodium 140 mmol/L, urea 16.8 mmol/L, creatinine 128 micromol/L, and blood glucose 6.5 mmol/L. Serum muscle enzymes were markedly elevated: creatinine kinase (CK) 328 U/L (reference values: 40–208), LDH 266 U/L (reference values: 115–255), BNP 607 pmol/L (normal up to 28.9), elevated liver function test with ALAT 125 U/L (reference values: 10–70), alkaline phosphatases 232 U/L (reference values: 35–105), and normal bilirubin 9 U/L (reference values: 5–25).  Table 1 describes the levels of CK, myoglobin, creatinine, and urea (the combination was stopped on 6th day after admission). Urine analysis showed no significant microscopic hematuria or proteinuria. The patient was also screened for autoimmune diseases: autoantibody p-ANCA (IgG) with medium positive result (nonconclusive), autoantibody c-ANCA (IgG) negative, and autoantibody ANA negative. These results made the differential diagnoses immunological vasculitis, granulomatosis with polyangiitis, and systemic lupus erythematosus (SLE) unlikely. The rheumatological differential diagnoses polymyositis and dermatomyositis were unlikely because of negative autoantibodies known to be associated to polymyositis and dermatomyositis (Table 2). A spinal tap was performed to rule out Guillain-Barré syndrome as differential diagnosis. The spinal tap showed no pleocytosis, normal protein, and normal glucose level in the cerebrospinal fluid. An MRI of the cervical spine showed a nonsignificant spinal stenosis at the C5/C6 level, which did not explain the symptomatology of the patient. Finally because of negative HMG-CoA reductase antibodies (IgG), associated to autoimmune statin induced rhabdomyolysis, this differential diagnosis was unlikely in our patient (Table 2). Therefore, considering the clinical development, the kinetics of the biochemical markers, and the medication history, we assume that the diagnosis of rhabdomyolysis as a consequence to combination therapy with atorvastatin and fusidic acid is highly likely. Treatment with atorvastatin and fusidic acid was halted on 6th day after admission and approximately 12th day since the combination started. Fusidic acid was changed to oral Co-amoxiclav 500/125 mg tid and the patient continued on atorvastatin and oral moxifloxacin 400 mg daily. The patient was treated with intravenous fluid and physiotherapy.

**Figure 1:**
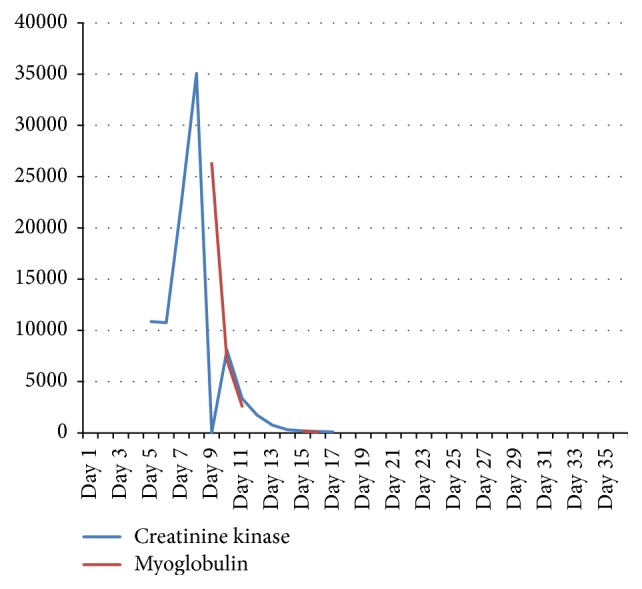
The graph shows the levels of creatinine kinase (CK) and myoglobulin in the *y*-axis and the admission days in the *x*-axis. The rapid fall and normalization of the CK and myoglobulin in our patient, after cessation of treatment with atorvastatin and fusidic acid on 6th day prior to admission, clearly suggest the diagnosis of pharmacokinetic interaction between atorvastatin and fusidic acid as cause of rhabdomyolysis.

The patient was discharged after 5 weeks with remarkable improvement of symptoms. When he left hospital the biochemical markers revealed normal muscle enzymes with CK 71 U/L, myoglobin 94 ng/L, and normal transaminases (Table 1 and Figure 1). It was decided not to treat the patient with fusidic acid in the future.

## 3. Discussion

Statins are HMG-CoA inhibitors (hydroxymethylglutaryl/coenzyme A) and they lower the cholesterol levels by competitively inhibiting the HMG-CoA reductase, the final pathway in cholesterol biosynthesis [4, 8]. The three statins (lovastatin, simvastatin, and atorvastatin) are metabolized by the cytochrome p-450 3A4 (CYP-3A4) isoenzymes, whereas pravastatin is metabolized in the liver by sulfation and fluvastatin is metabolized by the cytochrome 2C9 isoenzyme (CYP-2C9) [8].

Rhabdomyolysis is one of the complications of myopathies. The risk of myopathies and rhabdomyolysis is increased by the concomitant administration of fusidic acid with statins [4]. Plasma concentration of statins significantly increases by coadministration of fusidic acid and HMG-CoA reductase inhibitor [4, 7, 9]. The mechanism of interaction, whether it is pharmacodynamics or pharmacokinetics or both, is still unknown. Reports have shown rhabdomyolysis, including some fatal cases, in patients receiving combination of fusidic acid and its salts with oral anticoagulants such as warfarin, other coumarin derivatives, or anticoagulant; this may also increase plasma concentration of these anticoagulant agents, thus enhancing the anticoagulant effects with risk of bleeding. An interaction between fusidic acid and drugs biotransformed via CYP-3A4 system is suspected. Apparently the mechanism of this interaction is possibly mutual inhibition of metabolism [9]. However, recent studies suggest fusidic acid inhibits hepatic transporters and metabolic enzymes which may cause drug-drug interaction with statin coadministration [10].

The mortality rate associated with statin induced rhabdomyolysis is approximately 0.15 deaths per 1 million [4, 11] and it is considered as a rare side effect involving less than 0.1% of patients on statin treatment. The FDA database reports a mortality rate of 7.8% in patients with rhabdomyolysis [4, 12]. Cerivastatin was previously one of the most commonly implicated statin [4, 11]. Due to more than 100 fatal outcomes linked to rhabdomyolysis, it was withdrawn from the market in August 2001 [4, 12].

Factors increasing the plasma concentration of the statins increase the risk of rhabdomyolysis and hepatitis. These include concomitant use of lipid lowering drugs, hosts genetic factors, and drug interactions with other medications that are metabolized by the same cytochrome p-450 system of enzymes [7, 13]. Risk factors for these adverse effects include renal disease, hepatic dysfunction, diabetes, age above 80 years, and hypothyroidism [4, 14, 15]. The most common medications affecting statin metabolism, apart from fusidic acid, are fibrates (especially gemfibrozil), cyclosporine, warfarin, digoxin, macrolides, azole antifungals, calcium channel blockers, and amiodarone [2, 14–16].

The mainstay treatment of rhabdomyolysis is hydration and increasing diuresis. Diuresis protects the kidneys by diluting myoglobin in the renal tubules and hence prevents the toxic cast formation and also promotes the excretion [17]. Mannitol and urine alkalinization are also indicated in the treatment of rhabdomyolysis. To our knowledge there are no specific guidelines, but The American Academy of Clinical Toxicology and the European Association of Poisons Centre and Clinical Toxicologists have issued a paper recommending administration of a bolus of 225 mL of 8.4% sodium bicarbonate intravenously over 1 h, followed by additional intravenous boluses q. 1 h, to maintain pH between 7.5 and 8.5. Another regimen is to add 1–3 amps (50 mEq/50 cc) of sodium bicarbonate from 8.4% to 0.9% or 0.45% normal saline or 5% D5W [18]. In all regimes, high recommendations have been made to monitor the pH, serum potassium, and arterial pH hourly [7, 19].

## 4. Conclusion

This case report is highlighting the importance of early recognition and treatment of this rare but potentially fatal side effect, rhabdomyolysis, caused by use of the statin group of drugs, either separately or combined with fusidic acid. If statins are used to treat patients, fusidic acid should not be used. In conditions where life threatening infection is involved and fusidic acid is the only antibiotic option available, it is suggested that all statin treatments should be set on a halt, as long as the patient is receiving fusidic acid.

## Figures and Tables

**Table 1:** Muscle and kidney function biomarkers during the admission. The combination of atorvastatin and fusidic acid stopped at day 6 since admission.

Days (according to admission)	Creatinine kinase (normal 40–280 U/L)	Myoglobulin (normal 24–77 ng/L)	Creatinine (normal 60–105 micromole/L)	Urea (normal 3.5–8.1 mmol/L)
1	—	—	128	16.8
2	—	—	—	—
3	—	—	—	—
4	—	—	119	—
5	10865	—	108	—
6	10750	—	92	—
7	22520	—	134	—
8	35080	—	134	—
9	>20000	26300	100	22.2
10	8070	7110	85	21.8
11	3350	2590	74	19.5
12	1740	—	74	17.0
13	755	—	62	13.3
14	314	—	70	10.4
15	197	143	68	8.1
16	149	89	81	7.7
17	112	94	79	8.0
22	389	—	81	8.0
26	72	—	85	—
29	71	—	85	—

**Table 2:** Screening for autoantibodies known to be associated to polymyositis, dermatomyositis, and isolated statin (HMG-CoA reductase inhibitor) causing myalgia.

Antibodies	Results
P-Glycyl-tRNA synthetase-Ab. (IgG)	Negative
P-Jo 1-antibody (IgG)	Negative
P-Histidine-tRNA-ligase (Jo1)-Ab. (IgG)	Negative
P-Isoleucyl-tRNA synth. cytop.-Ab. (IgG)	Negative
P-MDA5-antibody (IgG)	Negative
P-Mi-2a-antibody (IgG)	Negative
P-NXP2-antibody (IgG)	Negative
P-Polymyositis (Ku)-A (IgG)	Negative
P-Polymyositis (PL-12)-antibody	Negative
P-Polymyositis (PL-7)-Ab. (IgG)	Negative
P-Polymyositis (SRP)-Ab. (IgG)	Negative
P-SAE1-antibody (IgG)	Negative
P-TIF1 y-antibody (IgG)	Negative
HMG-CoA reductase-Ab. (IgG) (HMGCR), normal reference < 20	<3
